# ‘That’s just weird’: A qualitative investigation into expert opinions on the difference between autonomous vehicles and humans deciding to kill

**DOI:** 10.1371/journal.pone.0332656

**Published:** 2025-11-05

**Authors:** Stephen R. Milford, Bernice S. Elger, David M. Shaw

**Affiliations:** 1 Institute for Biomedical Ethics, Basel University, Basel, Switzerland; 2 North-West University, Potchefstroom, South Africa; 3 Center for Legal Medicine, University of Geneva, Geneva, Switzerland; 4 Care and Public Health Research Institute, Maastricht University, Maastricht, the Netherlands; Universitat de Girona, SPAIN

## Abstract

Autonomous vehicles (AVs) are rapidly developing and in the process of being deployed on public roads. This has sparked extensive discussions on the ethics of AVs, particularly in collision scenarios. While much quantitative research has been done, little qualitative research has been conducted and none on the ethical opinions of experts who are actually responsible for developing, deploying, and regulating AVs on public roads. Making use of qualitative research methods, 46 experts were interviewed to obtain rich data on their ethical opinions of AVs deciding to kill human beings. Following thematic analysis, three overarching themes were identified: 1) Experts feel humans ultimately will be responsible for how AVs behave in collision scenarios. 2) AVs decisions lack important human characteristics such as ‘gut feelings, emotions, or intuition’ and would make uniformed decisions which do not reflect human decisions. 3) Some experts did have a preference for AVs making decisions in life and death situations. The paper ultimately concludes that experts who are responsible for how AVs are designed, deployed, and regulated hold complex opinions on the ethics of AVs making life and death decisions. Considering the public’s legitimate interest in this domain, far more work is needed to unify the ethical opinions of experts on the ethics of AVs in collision scenarios.

## Introduction

The impact of Autonomous Vehicles (AVs) will be beyond simply transportation [1]. From improved traffic flow and congestion [2,3] to better general access for the elderly or disabled [4], from environmental benefits [5] to an impact on public health [6]. Yet without a doubt the greatest perceived benefit is the anticipated radical reduction of road deaths [4,7,8]. Some estimates put this at a 90% reduction [9–11]. Considering that annually over 1.3 million people die on public roads, with 50 million more injured, this would be an incredible benefit [12]. Yet it is this point precisely that has captured the public’s attention in paradoxical ways. How can AVs reduce road deaths ethically? More than reducing human error [8] and improving safety [13], AVs may need to take action in life and death scenarios that could present them with moral dilemmas?

While some dispute that AVs will ever be faced with these scenarios [14–17], others argue that it is inevitable [18–22]. Consequently, it is important that we wrestle with the ethics of trolley-problem-like scenarios for AVs [23], if for no other reason than the fact that the public is deeply concerned with these issues [24]. Indeed, the public’s engagement in the ethics of AVs choosing to kill or save lives is extensive [25] and has sparked widespread debate in the ethics of AV killing and in trolley problems in general [7,26–31]. The ethics of machines killing human beings have been debated long before AVs, particularly in the context of Lethal Autonomous Weapon Systems (LAWS) such as military drones. Some argue that these autonomous killing systems could be good news as they reduce human suffering [32], while others doubt the morality of killing at a distance [33]. There is reason to argue that the public are generally against machines making moral decisions [34] with the EU calling for a moratorium on LAWSs [35]. In the words of Coeckelbergh:

There seems to be a fundamental asymmetry between humans and robots when it comes to their capacities to make good moral decisions, and on this basis we should ban any project that aims to have machines (e.g., drones) make decisions about life and death [36].

For many, the question of autonomous killing is connected not only to LAWS, but also to AVs [36–38]: ‘After all, transport accidents, healthcare practices, and abuse of personal data may affect people’s life as much as military operations’ [37]. Indeed, critical perspectives have emphasized the importance of understanding AVs not as autonomous moral agents, but as components in a sociotechnical system shaped by human and institutional decisions [39–41]. Questions of transparency, responsibility, and value alignment cannot be reduced to algorithms or logic trees. Instead, as Martinho et al. [42] and Lapaolo [43] argue, we must examine how AV development is guided by ethical assumptions embedded within industry practices.

Despite the growing critical normative literature [15,44,45], there remains a notable lack of empirical research into the perspectives of those directly responsible for AV development, deployment, and regulation. While studies such as Stilgoe (2021) explore safety and risk governance [46], and Martinho et al. (2021) review ethical themes emphasized by the AV industry [42], few studies gather rich, open-ended insights from developers and regulators themselves. This gap is significant: as Stilgoe and Mladenović (2022) argue, the politics of AV development hinge on expert assumptions about ethics, autonomy, and public acceptability [41]. It is these experts who are both directly and indirectly responsible for how AVs behave in life and death situations. Assuming that experts are in a particular strong position to make informed decisions, their attitudes are vital to understanding how AVs might, or should, behave in situations which involve the life or death of members of the public. The present study is the first of its kind and goes some way to filling this research gap.

## Methodology

### Sample and data collection

As part of the National Centre for Competence in Research (automation – Switzerland), the Proactive Ethical Approach to Responsible Automation (PEpp) project interviewed 46 experts between the 21/03/2022 and the 27/07/2023 about the ethics of AVs on public roads and in particular on the ethics of AVs making life and death choices. Participants were chosen who had a range of expertise either directly or indirectly related to AVs on public roads at academic, private, and public organisations. A total of 33 experts had direct experience in developing AVs, this included developing motion planning algorithms, working for universities and private organisations to develop AVs, acting in senior leadership positions within AV organisations (such as CEO or Chief Engineer), testing AVs on public roads for well-known AV companies, or being involved in implementing public trials of AVs. Ten experts had indirect experience such as programming or researching in the field of traffic management, robotic and automatic control systems, or control engineering for transportation applications. Three experts were involved in the regulation of AVs in Switzerland, the EU, and the USA at state level.

Following ethics approval from the University of Basel’s Ethics Committee, a semi-structured interview guide was developed informed by the literature on AV ethics and aligned with the study aims. The semi-structured format allowed flexibility for participants to elaborate beyond the guide while ensuring comparability across interviews. Questions were designed to focus on the ethics of AVs on public roads. This included questions of the role and responsibilities of experts as well as how AVs should be regulated. Of significance for the results presented here are interview questions that explored experts’ opinions of AVs making life or death choices compared to human drivers making these same choices. Questions were open-ended, with optional probes and scenario-based prompts (e.g., trolley-problem-like cases) to elicit nuanced reflections. All participants gave their written as well as verbal consent before being interviewed. Initially three pilot interviews were conducted to test clarity, flow, and timing; these were evaluated by researchers and the PI, and the guide was refined accordingly.. Purposive as well as snowball recruiting was undertaken to identify potential participants [47,48]. Interviews were undertaken in English, lasting approximately 1 hour with participant recruitment continuing until saturation was achieved, upon which 2 further interviews were conducted to confirm saturation [49]. Using an experimental ethics approach to qualitative research, experts were encouraged to go beyond simply stating a belief they had about a particular topic, rather they were encouraged to actively engage in exploring and justifying their opinions [50–52].

### Data analysis

All information permitting direct or indirect identification was removed from verbatim transcriptions of the interviews so as to anonymise all data (e.g., name, place of work, role etc.). Using MAXQDA – a well-established qualitative research analysis software program – transcriptions were subjected to reflective applied thematic analysis in order to analyse and highlight the significant thematic elements that arose from the data [53–55]. Researchers began by familiarising themselves with the data set through actively reading and re-reading the transcriptions while writing down initial ideas for codes and themes. Researchers proceeded to engage with open coding so as to inductively generate descriptive codes from the data. The first author initially coded three interviews after which a meeting was held with the research team and the PI to evaluate the quality of coding. Following this, the remaining transcriptions were coded with regular supervision and meeting with the PI. An extensive code tree was developed and agreed upon by the research team. Once the code tree was agreed, descriptive codes were sorted for themes. The research team reviewed the overarching themes created by the first author to agree the themes identified. In the last step the themes were interpreted and organised into coherent accounts that would support core narratives present in the data set. For more, see the companion articles reporting other findings on the research [56,57].

## Results

This study recruited 46 experts who had direct and indirect experience of developing, deploying, and regulating AVs on public roads. Each participant engaged in a semi-structured interview based on an interview guide. Interviews were not prescriptive, and participants were encouraged to speak freely. The interviewees were sensitive to the leadings of the participants. Following data analysis, three key themes were identified. These themes (and their sub-themes) emerged from the data and often transversed individual questions and participants. The results presented here are of the participants who spoke to these themes (in some cases to multiple themes). As far as possible we present data without interpretation or discussions.

### 1 Is AI Actually Making Decisions or ‘Acting for Someone Else’?

A large number (16) of experts noted that fatal decisions enacted by AVs are not the result of AVs themselves but determined by external factors (see Table 1). This concept emerged throughout the interview process and was not mentioned by the remaining participants. Within the cohort of participants that mentioned this theme, a number of experts referred to human programmers/designers as the ultimate source of decisions enacted by AVs during collision scenarios. For example, Participant 3 explicitly stated: ‘decisions are not made by AI, they’re made by the designers of the AI.’ Likewise Participant 2 did not ‘truly see a difference’ between a human and an AV acting in collision scenarios.

**Table 1:** Theme 1 Data Extracts.

AVs derive their decisions from external factors.	So, the decisions are not made by AI, they’re made by the designers of the AI. p#3 Innate objects [AVs] don’t have agency. So the decisions are not made by AI, they’re made by the designers of the AI. (p#5) I mean in the end, it’s a bit like animals, right, you can get killed by a lion, and it has an impact on you and the people around you, but you don’t see them as ethical necessarily. Like, they don’t have moral rules, right. So, AI could be considered in the same way, even though they make decisions. (p#6) If an artificial intelligence grows in an environment that creates something and this environment is made by humans then there’s a similar responsibility. (p#8) It’s always the end of the day, it’s programmed to do something. p#9 Somebody human must have been in charge at some moment in the decision process. Not necessarily in the last milliseconds. p#11 Now, that AI, how it’s gonna be trained? So, which, which people behaviour it will replicate? Moral people, immoral people, average people, cross-cultural set-up… some average human all over the planet? So, I have very big troubles understanding how this will look like. p#13 It’s a machine, right, an algorithm. (p#25) Systems at some point [are] always developed by human developers. p#27 Well, it’s still a human that decided to take the life, right, like, it’s, it’s still humans who are designing this. p#33 The AI is making the decision? Well, it’s not… it’s still relying on the programs that it’s been given. p#42

There was a sense among some participants that if AV decisions were merely the product of exogenous factors (including human programmers) then there is a question of who, or what, forms the basis for the ethics that guide decisions made by AVs in collision scenarios? For example, Participant 13 had ‘very big troubles understanding’ which human behaviour the AV would replicate. Participant 10 was unsure of who, or what morals, underpin an AV decision and, therefore, stated that they ‘would be against’ AVs killing humans in a dilemma scenario. By this they meant AVs should not be involved in the killing of human beings at all – although no clarification was given as to how that might work in practice.

Other participants in this group noted both human and non-human influences. Participant 8 argued that human ethical decisions are determined by the environment in which respective humans grow up – their upbringing, cultural and other ‘exogenous factors.’ AIs are likewise determined: ‘an artificial intelligence… also emerge[s] from some data or something that has been fed to this, to this intelligence.’ These participants felt that it was not the AV itself that determines the ethics involved in collision situations, but rather these exogenous factors (data provided, sensor equipment etc.). This environment naturally also includes humans. To Participant 6 ‘the AI is acting for someone else.’ Yet there was a tension in Participant 6’s position in that he/she also understood the AV as being neither ethical nor unethical in the same way that an animal acts neither morally nor amorally. To Participant 6, an AV killing a person could be compared to a lion killing a person: ‘you don’t see them [lions or AVs] as ethical necessarily.’

### 2 Should AVs Make Life and Death Decisions

The notion that AVs merely reflect exogenous factors (including human programmers) lead a number of experts to question the suitability of AVs having autonomy in ethically difficult scenarios such as road collisions. This larger theme emerged from a number of sub-themes associated with a wider concern about AVs making life and death decisions.

#### 2.1 AVs Lack Key Human Characteristics.

Some participants expressed concerns about AVs making decisions that could ultimately kill a human being because these AVs lacked what some participants felt were important human characteristics necessary for such decisions (see Table 2). For example, ‘gut feeling, emotion or empathy’ (p#3). Participants 9, 22, 25, and 31 asked if an AI would have ‘some sort of moral compass that aligns with [humans]’ (p#9) but found that ‘difficult to image’ (p#9). Indeed having a moral compass in a machine, according to Participant 9, would be ‘just weird.’ Participant 22 argued that it would be impossible for an AI to make moral decisions because ‘it will take purely logic [sic]’ decisions; that is, decisions without emotion. They went on to argue that for these rational decisions to be perfect, the AV would need ‘the whole knowledge of the universe’ but this would never be possible ‘in this universe.’ We speak more about this in the next sub-theme.

**Table 2:** Theme 2.1 Data Extracts.

Whose morals are we using?	What is the moral that that the machine is following? And that’s something we, we don’t know, right? That’s something we, we don’t have control on… And without those answers I would, I would be against what you say [a car killing a human in a dilemma situation]. p#10 There [are] also human biases which affect[s] machine… let’s say, previous decision that could be biased, and then they [are] picked by this algorithms and then deployed, and then, of course, this reinforces things. p#2 They usually are less, perceptive about this gut feeling, emotion, or empathy. Right?… so I think that is more, the main difference would be about empathy with what you have in front of you. p#3 That is just weird [AVs making ethical decisions]. Maybe because I’m sceptical that it will ever have more, more of a human like moral intuition. Maybe if I see evidence… So I do not trust such a technology to make more, to have a bit more of human intuition. But I don’t know, ask me again in many years. p#9 I think that’s the main difference. You and me, we have a moral compass clearly. p#25

#### 2.2 Uniformed Decisions are not Desirable.

While Participant 22 struggled to imagine a world in which an AV that was able to make a purely logical decision, others could imagine this with conflicting opinions on its value (see Table 3). Participant 5, linking to the idea that AV decisions ultimately come from human programmers, argued that programming AVs in dilemma scenarios would be good for society as it would ‘force us to really specify in code… what are our principles,’ and this would remove ‘any inconsistencies of human reasoning.’ While there were those who agreed that reflective thinking was better than reflexive thinking (see theme 3.1 below) others argued that a purely logical AV would make decisions that are ‘uniforminized [sic]’ (p#16). To Participant 17, ‘if we design an AI it is just the one algorithm. Every AV will do one thing.’ This would not be a ‘uniform distribution’– referring to the Bell curve – but ‘more like a point load distribution.’ Participant 17 felt we would lose something here as ‘everyone [humans] will have a different decision’ in such dilemma scenarios. Participant 17 saw this as a positive aspect of human drivers. To Participant 20, AVs that act in uniform ways, are acting ‘completely oppositely to human[s]…[who]have emotion.’

**Table 3:** Theme 2.2 Data Extracts.

Avs and rational uniformed decisions	There’s still something where it’s all uniforminized [sic] in a certain way for example, right? p#16 [AVs act] completely oppositely to human[s]. Human[s] have emotion… [are] not parameterized, it’s not trained and so on. p#20 It cannot decide because… there is no moral decision, it will take purely logic… even if you can program it, it will be a programme, it will always look the same. Unless we are able to provide the whole knowledge of the universe and let it judge… but this is not possible. Not in this universe. We need another one. p#22 Yes, there is clearly a difference [between an AV and human driver] just me telling you like the two key arguments for me deciding doesn’t mean that within that second, I come up with 30 other ideas why I choose Person A or B? I think this intuition [a] machine will never have. p#25

#### 2.3 Black Boxes.

A larger sub-theme, linked to the question of data transparency, was identified (see Table 4). Among the group of experts who expressed a more pessimistic view, i.e., they thought AVs should not make decisions in dilemma scenarios, a number of experts (6) expressed a concern about the transparency of AVs. This was connected with the theme of AVs not ultimately making their own decisions (theme 1 above). For example, Participant 3 stated that the opacity of AV decision making questions the foundations of those decisions, while Participant 18 argued that it would be hard to trace the decisions of an AV to see who ‘forced that decision’ – referring to the AV programmers and developers.

**Table 4:** Theme 2.3 Data Extracts.

Transparency is important	Whereas for now at least, we have no clue how AI works, even the non-general ones. So, it’s hard to improve on it. p#6 If we can follow the decisions that, why the AI has taken, you know, such a decision, if we actually [get] inside into that, I would actually prefer the AI. p#7 It’s very easy to trace back what people do to who did the, make the decision. If things get automated, then it’s a little bit more difficult to trace it back to… who’s decision, right?...[with transparency] I don’t see a problem on delegating it. Delegating this [dilemma decision] to automated systems. p#8 It can be that there is some mistake in the data. p#11 Cause as a human I make a conscious decision, whereas for an artificial intelligence it will be very hard to trace back why it made that decision and who forced that decision. Was it somebody in this ethics committee, was it the person coding it, was it, I don’t know what. p#18 I would [prefer] the machine if I [am] inform[ed] about how the machine would act. p#20 I mean it’s very difficult to guarantee that these decisions are unbiased [especially] if it’s an ethical decision. p#21

Even among the group that felt AVs would be preferable to humans, a few participants only considered this possible if transparency could be guaranteed. For example, Participants 7 and 20 argued that if an AV decision-tree could be mapped, and we could know why a decision was taken, they ‘would actually prefer the AI’ (p#7) to a human driver. To Participant 8 this represented a ‘delegated’ decision which they would be happy with. Participant 11 put a further caveat on this criterion by arguing that the decisions of the AV must be shown to be ‘the best option compared to all other possible ones.’ Participant 11 did not elaborate on how the ‘best option’ would be measured.

#### 2.4 Responsibility Must Remain with Humans.

Consequently, a number of participants felt it was important for humans to remain in the decision-making loop and ultimately take responsibility for the death of a human being (see Table 5). To Participant 4, even if the AV might make a ‘better’ (p#14) choice, ‘we are not even ready’ to accept this outcome, nor will we be in the future. While Participants 11 and 21 felt that humans should always be involved where moral or ethical decisions are being made – even if this is at the programming level. Participant 11 argued that even in other spheres where machines are used to kill people, such as military applications, ‘the end [is] screen[ed] by somebody human.’ I.e. the end result is evaluated by a human operator before being approved. Therefore, ‘there must be a human…that either approve the rule or approves the action’ (p#11) while Participant 21 argued that they would prefer if a user could take over at any time.

**Table 5:** Theme 2.4 Data Extracts.

Humans in control	I just think that we are not even ready, I’m not sure we will be ready to let an artificial intelligence making this kind of choice. p#29 The end [is] screen[ed] but somebody human, and only if there are completely accepted procedures then the machine can take decisions…. there must be a human…that either approve the rule or approves the action” p#11 [I] would prefer that anyone of the users [AV users] should at some point be able to say, ok, exclude the system and they do things by [themselves]. p#21

### 3 A Preference for AV Decisions

#### 3.1 Human ‘Reflex’ Decisions are Not Optimal.

A number of experts ‘prefer[ed] AI making the decisions’ (p#38) in ethically problematic scenarios if certain conditions were met (see Table 6). One aspect of this theme was that humans – under time constraints – do not make good decisions. Participant 5, for example, who initially stated that human programmers ultimately made AV decisions went on to argue that ‘in the future… the decisions made by an AI will probably be better than the decisions made in haste by a human under stress.’

**Table 6:** Theme 3.1 Data Extracts.

Preference for AV decisions	[An AV] can grab a lot of data…so it can make decisions quicker, faster, and even better than human beings. p#14 I would rather have the AI make the decision because I believe that technical systems in those situations can probably produce a better understanding of the world. Okay, that said, I don’t know whether that’s true today, but I think when AVs will be generally deployed… they have a better understanding of the world… So I would trust the AI to make that decision better. That could be a real decision, and the human is just a reflex. I don’t think that’s a real decision. p#24 Actually having an almost sentient vehicle is always going to be a vast improvement. Because it don`t [sic] get tired, they’re always vigilant, they remember everything, and they can share what they’ve learned and what they`ve experienced with other vehicles and other communities. p#35 I would probably trust a computer AI system to make a faster more logical decision than potentially a human that is much more reflex driven. Now, that’s assuming we’ve reached a spot where AI is acting as fast or not faster than the human synapses in the brain to be able to make those decisions p#40

Participant 14 claimed that a person who ‘has no time to think’ and thereby makes an ‘instant reaction’ has not made an optimal decision. To them, AVs will make better decisions because they can make decisions faster than a human being. Participant 24 argued that humans make reflexive decisions which are not ‘real decisions,’ and that machines would have a better understanding of the world in dilemma scenarios because of their sensors and access to wider knowledge. While Participants 35 and 40 argued that an AV’s decision is a ‘vast improvement’ (p#35) on a human who gets tired and is not always vigilant. The participants who preferred AV decisions, however, acknowledge that one had to ‘assume’ (p#40) AVs were quicker than human beings, which they doubted were ‘true today’ (p#24) as ‘at the moment we are very far from that’ (p#14).

#### 3.2 AV Decisions Offer Advantages.

There were two participants who highlighted the advantages of AV decisions (see Table 7). Participant 45 presented a paradox by arguing that an important ‘human psychological trait’ is to blame someone. Yet an AV could not be blamed as it was merely a machine. On the other hand they noted victims’ families – and the community as a whole – may better accept a fatal incident from an AI than a human because there is no ‘face to blame.’ For them, it would be easier to accept an AV killing a person, than another person doing the killing.

**Table 7:** Theme 3.2 Data Extracts.

AV Decisions Offer Advantages	[An AV will] never say, oh yeah, I’m really sorry it was my fault. But you know, nine times out of 10 you do look to someone else to blame. That’s a human psychological trait…. Some people might prefer it because there isn’t a face to blame. They might prefer [the decision to be made by an AV] that it’s easier for them to accept that it was a machine, not human. p#45 [Say] we would like that behaviour to change [speeding around a school] … you can try and do that with all the drivers in that area, it will take you 40 years… Or you can go to us as an operator and… we’ll do a software update, and it’ll be implemented on all the vehicles from tomorrow… which option would you prefer? p#39

Participant 39 argued that AVs give us the opportunity to immediately change road behaviours without having to retrain the entire population. For example, when we chose to reduce the road speed, or open a new school in an area. Changes to speed and behaviour will ‘be implemented on all the vehicles from tomorrow.’ Consequently, we can update the way life and death decisions are made instantly and do not have to wait for human beings to learn new ways of approaching challenging situations.

#### 3.3 AVs Should be Held to a ‘Higher Standard’.

For Participant 29 the ability to make quicker decisions based on more information necessitated that AVs be held to ‘a higher standard’ when evaluating the ethics of their decisions (see Table 8). Similarly, Participant 5, argued that the pre-trained, rational response of an AV represents a form of ‘intent’ to kill that is not present in reflexive thinking.

**Table 8:** Theme 3.3 data Extracts.

A Higher Standard	There is intent behind it [the decision], it’s not just sort of an accident in that sense. p#5 if they do have, if this sort of like the general AI, they, you know, have all the information, they’re able to make [decisions] super quickly, then I think it does become important to, to sort of evaluate them on a, sort of a higher standard because they do have more, more time, and more forethought to, to make those decisions. p#29

## Discussion

The results presented above paint a picture of non-uniformity among expert opinions. While many considered AVs to be merely acting out the instructions of human programmers, and therefore, not moral agents, others engaged with the idea of an AV making a life-or-death choice. For some, this was problematic as AVs lacked key human characteristics and would make non-transparent uniformed decisions that were ultimately decidedly different to how human beings make decisions. For others, enabling AVs to make decisions offered certain advantages, notably the reflective (as opposed to reflexive) nature of these decisions; the possibility for society to better come to terms with tragedy if it were the result of a machine, and the ability to immediately change road behaviours. Nevertheless, while it is possible that AV decisions should be preferenced, there was a strong sense that their decisions should be held to a high standard. Let us discuss each of these opinions in turn.

### AVs are not moral agents

There is a challenge when speaking about an AV ‘choosing’ or ‘deciding’ to act. These terms are often associated with human beings and strongly connected to philosophical discussions around intentionality or moral agency [58,59]. Traditional algorithms cannot strictly be said to ‘choose’ or ‘decide’ a course of action in that they merely adhere to their programming. Modern AVs, however, are increasingly driven by AI which often acts as a black box with an opaque decision tree. During this research we deliberately did not define concepts such as ‘decision’ or ‘choosing’ as we were interested in the opinions of participants as to whether there is a difference between an AV’s actions and a human’s actions. Thus, we left it for participants themselves to interpret terms such as ‘choosing’ or ‘deciding’ when giving open-ended questions. The results of this research indicate that AV experts have divergent opinions of the challenges posed by AVs taking human lives compared with human beings making the same choices.

As expected some experts noted in theme 1 above raised questions about the nature of these decisions. That is to say, are such decisions actually the result of an AV itself, or merely of its programming and consequently those who have programmed the AV. Perhaps more problematic, some noted that the decisions may be the result of the training data or context of the transportation environment rather than a human programmer. While this was not explicitly stated, it is implicit among such experts that an AV is not itself an autonomous moral agent. A moral agent might be defined as an autonomous subject with a reasonably fixed idea of what is good and whose free will enables them to determine the principles of their own actions [7,60,61]. However, for many of our participants, AVs at present (driven by current AI as they are) cannot be understood as autonomous subjects. Therefore, it is incoherent to consider an AV making a ‘decision’ in the same way as a moral agent makes a decision. To the experts that spoke to theme 1, AVs remain merely machines that enact decisions based on exogenous factors. This is ultimately the current position of the EU on autonomous systems. The EU has stated that: ‘Since no smart artifact or system – however advanced and sophisticated – can in and by itself be called ‘autonomous’ in the original ethical sense, they cannot be accorded the moral standing of [a human person]’ [62].

In this sense, ethical implications of AVs are not reducible to instantaneous decision-making during a crash, but rather extend upstream to the institutional and technical infrastructures in which AVs are embedded. This aligns with the argument by Stilgoe and Mladenović (2022) [41], who emphasize the politics of AVs—that their development is never ethically neutral but reflects decisions about concern mundane issues of safety, accountability, and public trust that are made beyond an individual AV itself. Marres, for instance, shows how street trials of AVs are not merely technical tests but public experiments in participatory ethics, where nothing happening can be as meaningful as something going wrong [40]. Similarly, Ganesh critiques the “ironies of autonomy” in how the language of independence masks deeply relational and embedded ethical decisions [39]. These perspectives are echoed by several experts in our study, who emphasized that AVs are never truly autonomous but act within a framework of delegated human responsibility.

The lack of moral autonomy led some experts in this study to question the moral framework underpinning AV decisions that results in the death of a human being. This begs the question: Whose moral frameworks should be used as the guiding frameworks for how AVs make decisions? This discussion is taken up in the literature as a number of authors wrestle with the vague notion of AVs having to mimic human driving behaviour and ethics [38,63–65]. However, what is not stated in the literature is the notion that it is AV development experts themselves who are ultimately responsible for designing, programming, and implementing AV decision-making algorithms (DMAs). Some experts who participated in this study were keenly aware that AVs will not be making ethical decisions themselves but rather were heavily influenced by experts such themselves. As such they were conscious of the significant questions surrounding who exactly will be guiding these decisions. It should be noted that results reported elsewhere from this same study have shown that AV experts have poorly developed moral frameworks [57].

### AV decision making is markedly different to human decision making

Theme 2 presented an interesting tension within some experts in this study that is worthy of note. Some of the experts who spoke to theme 2, also spoke to theme 1 (e.g., p#25 and p#9). On the one hand these experts understand AV decisions to be the direct consequence of exogenous factors, primarily of which is their human programmers. Yet on the other hand, these experts also expressed concern about the marked difference between an AV DMA and a human’s decision-making process. This concern implies that these experts believed that AVs are indeed making decisions and that these decisions are based on different processes/basis to those of a human being.

For the experts who spoke to theme 2.1 of this study, the apparent lack of human-like characteristics such as ‘gut feeling, emotion or empathy’ (p#3 also present in p#20) present in AV decisions was problematic for the ethics of AVs killing human beings. Reference to human-like values as the basis of ethical decision making is markedly different in our study to how it is presented in the literature. In the literature very rarely – if ever – does reference to such characteristics arise. Even among authors who argue that AVs should mimic human values, notions of ‘gut feelings’ emotions or human intuition are not the basis of these vales [38,65]. In fact, authors such as Frank et al. [63] would argue that such emotionality can lead to a bias in human values and presents a social challenge to producing a universal moral code for guiding AV DMAs. Rather than referencing characteristics such as emotion, much of the discussion in the literature revolves around higher-order reflective and rational ethics such as utilitarianism, deontology, or the social standing of one person versus another [25,63,65]. Even in discussions involving what has come to be known as the ‘ethical knob’ [66], whereby individual humans can choose what ethics to impose on their individual cars, the questions of what values should be included as options on this setting are often highly formal as opposed to practically rooted in human emotive characteristics [25,67–69].

Yet, the experts in our study, indicated that a lack of these apparent human characteristics would be a challenge to the ethical integrity of an AVs decision to kill a human being. This is in opposition to the views of authors such as Frank et al. [63]. That is to say, should AVs be considered responsible for their decisions, their lack of empathy or emotion would actually question the morality of these decisions. To add to the paradox, participants such as Participant 9 argued that AVs that did have these types of characteristics would be ‘weird’. For an expert such as Participant 9, an AV’s ethical decisions are ultimately merely programmed by a human being, yet they also argued that the AV’s decision-making process is devoid of certain human characteristics (‘empathy, gut-feeling, intuition, or emotion’) and if the AV ever did have such a human intuition, it would be ‘just weird.’

The paradoxes did not stop there. A key concern, expressed in the literature and the experts who took part in our study, is that of the black-box problem (sub-theme 2.3). This problem applies to AVs specifically and to AI generally. Transparency is fundamental to trust, yet AI often presents a challenge to this transparency [70–73]. We regularly cannot trace the decision-making processes made by an AI. The experts in this study stated that the decisions of the AV were ultimately those of human programmers and at the same time expressed concern that an AVs decisions were not transparent. That is to say, it is difficult to trace the decision-making process of the AV to identify how it made a specific decision, and this in itself is an ethical challenge according to some of the experts of this study. The experts in this study hid not seemed to reconcile these two apparent contradictions: that AV decisions stem directly from human programmers, yet an AVs DMPs are opaque and difficult to trace.

One notion that was significant for the topic of the ethics of AVs making life and death decisions, as opposed to human beings making these decisions, was uniformity (sub-theme 2.2). For some of our experts, uniformity in ethical decisions was not considered a desirable attribute. Some experts argued that there is something intrinsic about the non-uniformity of human decisions that adds to their moral integrity. The exact underlying assumptions are difficult to gauge. For some, like Participant 17, it would be against the normal distribution of ethical decisions (if such a thing were to exist) if all decisions were the same. To Participant 20, uniformity would be opposed to the types of human characteristics mentioned by them and others: such as empathy, gut-feeling, intuition, or emotion (sub-theme 2.1). The response from this participant is significant as it indicates that some experts transfer non-ethical frameworks to those aspects of their work that concern ethics. This may indicate a form of modified ethical deliberation. Participant 20 seems to indicate that heterogeneous decision making is an observed phenomenon that is ‘normal’ and desirable. Elsewhere we have shown that experts do not have a well-developed ethical framework [57]. In Participant 20 their non-ethical training (statistical analysis) is being used to dictate desirable behaviours for AVs in ethical situations. However, one would be hard pressed to find a trained ethicist who would approve of a normal distribution function as the basis for ethical decisions.

This aversion to uniformity is very rarely spoken of in the literature. On the contrary, Coeckelbergh [74], for example, notes that some may argue that a machine would make better moral decisions because they do not rely on emotions which, in moral philosophy, are held to be problematic because of their unpredictable nature. However, Coeckelbergh goes on to argue that a moral agent requires a certain type of reasoning (including feelings) for moral deliberation. Consequently, moral decision making requires a particular kind of knowledge ‘an embodied kind of knowledge’ [74] that is sensitive to contexts and situations, developed through practical wisdom and lived experience. While Coeckelbergh acknowledges the necessity for certain human characteristics, non-uniformity is not mentioned.

The experts presented in theme 2 of this study, however, emphasised both the ethical challenges to the inconsistencies of human reasoning, and the ethical value of non-uniformed outcomes. There is an implicit belief in much of the literature that consistent ethical decisions across AVs are desirable. From a technical perspective, consistency is often equated with fairness and safety. However, from the view of some experts of this study, human non-uniformity — our capacity for context-sensitive variation — is a moral asset. This insight parallels arguments by scholars like Marres [40] and Ganesh [39], who emphasize the value of ambiguity and local responsiveness in ethical governance. Yet there remains a clear paradox in arguing on the one hand that programmers are morally responsible for AI decisions, while also arguing against uniformity and in favour of emotion in making those decisions: by definition, programmers are not present ‘on the road’ and will (hopefully) endeavour to follow some type of objective and uniform ethical code or framework. How exactly one creates non-uniformed decisions without emotions or feelings is a challenge that cannot easily be resolved without relying on pure randomness – which itself would be ethically problematic.

Thus, experts who spoke to this theme of the study called for human beings to be ultimately in control of AVs when it came to life and death situations. While this need to exercise meaningful human control is well-documented in the literature [75], the basis for this control is markedly different in our study than in well-established frameworks such as in the EU. As we have noted, the EU statement on autonomous systems being involved in killing humans has argued that at present a machine cannot be a moral agent. However, this statement goes on to express that even if it is technically conceivable for a machine to be a moral agent, the basis of human rights – human dignity – ‘implies that meaningful human intervention and participation must be possible in matters that concern human beings and their environments’ [62]. In our study, however, the experts presented in theme 2 were concerned about an emotionless, opaque, uniformed process as the basis of deciding to kill a human being.

### A preference for AV decisions

Among the experts who were of the view that AVs would make better decisions (theme 3), opinions were shared that both converge and diverge from the literature. The experts shared the well-known advantages of AIs generally; the ability to process substantial amounts of data and to make rapid decisions. To these experts, these facets of AI imply that an AV would make better decisions than human beings. What is not mentioned in the literature, at least to our knowledge, is the pastoral advantage of having an AV kill a human being. That is to say, that there is no ‘face to blame’ (p#45) in the tragedy of an AV taking a human life was a novel idea presented here. It is an open question as to whether this is indeed a pastoral advantage or not. Nevertheless, the perception held by Participant 45 was interesting and may well hold true for some.

## Limitations

A number of limitations should be noted. Frist, this is an international study and many of the participants did not speak English as their first language. However, it should be born in mind that many of the non-English participants were engaged in highly technical activities and were therefore highly educated and working in primarily English environments. Thus we feel that this is not a significant limitation, and their meanings are accurately reflected in this paper.

A second limitation is that the participants of this study are primarily from, or working in, Western contexts, mainly in Europe and the USA. Thus the opinions and ideas expressed by participants come from a particular cultural perspective. Consequently, it would be difficult to extrapolate from this data the opinions of experts from other regions or cultural groupings. While the data may not be generalisable, this does not diminish its significance. In fact, as with the methodology employed (qualitative research) the ultimate goal is not to produce generalisable results, but to provide rich descriptions of a phenomenon, in this case the opinions of experts on AVs making life or death decisions in comparison to human drivers making these same decisions [76,77].

## Conclusions

AVs present numerous ethical challenges one of which is the ethics of autonomous killing. While this has been debated in other fields, such as LAWS, it is also pertinent to AVs. There is evidence that the public is interested and concerned with this aspect of AVS and if AVs are to be widely adopted, the ethical concerns must be addressed. While there has been normative and empirical research conducted in this field, no research has considered the ethical opinions of those who actually regulate, develop, and deploy AVs on public roads. This is a noteworthy gap in the research, as it is these experts who are directly and indirectly responsible for how AVs behave in life and death scenarios. Consequently, the present research project, and the findings presented here, are significant. The findings of this qualitative research demonstrate that experts hold a diversity of perspectives. While some understand AV decisions to be nothing more than the consequence of exogenous factors (including human programmers), others do indeed perceive AVs making decisions and that these decisions are fundamentally distinct from the decisions taken by human drivers.

Some experts are of the opinion that AVs would make better decisions than human drivers in life and death situations. Unlike human drivers, AV decisions would be non-reflexive. They would be deliberate, intentional, logical, and thought through. Furthermore, it might be easier for the human community to come to terms with the consequences of a life and death decision made by a machine because it would be difficult to blame a machine in the same way one blames a human being. Nevertheless, these decisions need to be held to a higher standard than human decisions.

On the other hand, many experts expressed concerns about AVs making life and death decisions for a number of reasons. AVs apparently lack key human characteristics such as feelings, emotions, or empathy, and would make purely logical decisions. While the decisions may be based on pure logic, according to participants who question AVs ability to make better decisions, this itself presents challenges. First, the transparency of AV decisions is still a key ethical issue as the ‘black box’ nature of AI is well-known to those developing this technology. Second, the uniformed nature of AV decisions is problematic. To many of the participants of this study, there is something of value in the non-uniformed nature of human decisions. That is to say, the breadth of human responses is a valued aspect of human drivers.

However, it should be born in mind that many participants of this study felt that any decision made by an AV would ultimately reflect the decisions of human developers. To these participants, the AV is merely a tool and, therefore, could not be held accountable for its decisions. AVs do not represent moral agents. For many participants this was simply the nature of AVs, while for others this was a desired reality. Those that questioned the morality of a machine making life and death decisions, expressed a strong desire to retain a high level of meaningful human control [75]. This, however, presented a paradox as participants desired non-uniformity in decisions, but also uniformed control.

This research has demonstrated that those who are responsible for the regulation, development, and deployment of AVs on public roads do not have a unified opinion on the ethics of AVs making life and death decisions. While some feel that AVs will make better decisions, for most of the experts in this research there were fundamental questions about the ethics of allowing an AV to make these decisions, the types of decisions AVs will make, and the role of human beings in influencing these decisions. Considering the publics’ legitimate interest in this domain, far more work is necessary on the ethics of AV decisions in life and deal scenarios.

## References

[1] Othman K. Exploring the implications of autonomous vehicles: a comprehensive review. Innov Infrastruct Solut. 2022;7(2). doi: 10.1007/s41062-022-00763-6

[2] Milakis D, van Arem B, van Wee B. Policy and society related implications of automated driving: A review of literature and directions for future research. Journal of Intelligent Transportation Systems. 2017;21(4):324–48. doi: 10.1080/15472450.2017.1291351

[3] Szele A, Kisgyörgy L. Autonomous vehicles in sustainable cities: more questions than answers. WIT Trans Ecol Environ. 2018 [cited 22 Aug 2023]. doi: 10.2495/sdp180611

[4] Resnik DB, Andrews SL. A precautionary approach to autonomous vehicles. AI Ethics. 2024;4(2):403–18. doi: 10.1007/s43681-023-00277-6 38770187 PMC11105117

[5] Kopelias P, Demiridi E, Vogiatzis K, Skabardonis A, Zafiropoulou V. Connected & autonomous vehicles - Environmental impacts - A review. Sci Total Environ. 2020;712:135237. doi: 10.1016/j.scitotenv.2019.135237 31927439

[6] Sohrabi S, Khreis H, Lord D. Impacts of Autonomous Vehicles on Public Health: A Conceptual Model and Policy Recommendations. Sustainable Cities and Society. 2020;63:102457. doi: 10.1016/j.scs.2020.102457

[7] Etienne H. The dark side of the ‘Moral Machine’ and the fallacy of computational ethical decision-making for autonomous vehicles. Law, Innovation and Technology. 2021;13(1):85–107. doi: 10.1080/17579961.2021.1898310

[8] Evans K, de Moura N, Chauvier S, Chatila R, Dogan E. Ethical Decision Making in Autonomous Vehicles: The AV Ethics Project. Sci Eng Ethics. 2020;26(6):3285–312. doi: 10.1007/s11948-020-00272-8 33048325 PMC7755871

[9] Fagnant DJ, Kockelman K. Preparing a nation for autonomous vehicles: opportunities, barriers and policy recommendations. Transportation Research Part A: Policy and Practice. 2015;77:167–81. doi: 10.1016/j.tra.2015.04.003

[10] Airbib J, Seba T. Rethinking transportation 2020–2030: The disruption of transportation and the collapse of the internal-combustion vehicle and oil industries. RethinkX: Rethink Transportation. 2017. Available: https://static1.squarespace.com/static/585c3439be65942f022bbf9b/t/591a2e4be6f2e1c13df930c5/1494888038959/RethinkX+Report_051517.pdf

[11] Gao P, Kaas H-W, Wee D. Automotive revolution – perspective towards 2030 | McKinsey. In: McKinsey & Company [Internet]. 2016 [cited 17 Aug 2023]. Available: https://www.mckinsey.com/industries/automotive-and-assembly/our-insights/disruptive-trends-that-will-transform-the-auto-industry/de-DE

[12] WHO. Global status report on road safety 2018. Geneva: World Health Organization; 2018. Available: https://www.who.int/publications-detail-redirect/9789241565684

[13] Lim HSM, Taeihagh A. Algorithmic Decision-Making in AVs: Understanding Ethical and Technical Concerns for Smart Cities. Sustainability. 2019;11(20):5791. doi: 10.3390/su11205791

[14] Lundgren B. Safety requirements vs. crashing ethically: what matters most for policies on autonomous vehicles. AI & Soc. 2020;36(2):405–15. doi: 10.1007/s00146-020-00964-6

[15] Davnall R. The Car’s Choice: Illusions of Agency in the Self-Driving Car Trolley Problem. Artificial Intelligence. Brill | mentis. 2020. p. 189–202. doi: 10.30965/9783957437488_013

[16] Holstein T, Dodig-Crnkovic G. Avoiding the intrinsic unfairness of the trolley problem. Proceedings of the International Workshop on Software Fairness. New York, NY, USA: Association for Computing Machinery; 2018. p. 32–7. doi: 10.1145/3194770.3194772

[17] Hansson SO, Belin M-Å, Lundgren B. Self-Driving Vehicles—an Ethical Overview. Philos Technol. 2021;34(4):1383–408. doi: 10.1007/s13347-021-00464-5

[18] Kopecky R, Jirout Košová M, Novotný DD, Flegr J, Černý D. How virtue signalling makes us better: moral preferences with respect to autonomous vehicle type choices. AI & Soc. 2022;38(2):937–46. doi: 10.1007/s00146-022-01461-8

[19] Nyholm S, Smids J. The Ethics of Accident-Algorithms for Self-Driving Cars: an Applied Trolley Problem? Ethic Theory Moral Prac. 2016;19(5):1275–89. doi: 10.1007/s10677-016-9745-2

[20] JafariNaimi N. Our Bodies in the Trolley’s Path, or Why Self-driving Cars Must *Not* Be Programmed to Kill. Science, Technology, & Human Values. 2017;43(2):302–23. doi: 10.1177/0162243917718942

[21] Wang Y, Hu X, Yang L, Huang Z. Ethics Dilemmas and Autonomous Vehicles: Ethics Preference Modeling and Implementation of Personal Ethics Setting for Autonomous Vehicles in Dilemmas. IEEE Intell Transport Syst Mag. 2023;15(2):177–89. doi: 10.1109/mits.2022.3197689

[22] Fleetwood J. Public Health, Ethics, and Autonomous Vehicles. Am J Public Health. 2017;107(4):532–7. doi: 10.2105/AJPH.2016.303628 28207327 PMC5343691

[23] Keeling G. Why Trolley Problems Matter for the Ethics of Automated Vehicles. Sci Eng Ethics. 2020;26(1):293–307. doi: 10.1007/s11948-019-00096-1 30830593 PMC6978292

[24] Gill T. Ethical dilemmas are really important to potential adopters of autonomous vehicles. Ethics Inf Technol. 2021;23(4):657–73. doi: 10.1007/s10676-021-09605-y 34248401 PMC8253245

[25] Awad E, Dsouza S, Kim R, Schulz J, Henrich J, Shariff A, et al. The Moral Machine experiment. Nature. 2018;563(7729):59–64. doi: 10.1038/s41586-018-0637-6 30356211

[26] Dewitt B, Sahlin NE. Policy flaw in moral machine experiment. Nature. 2019;567:31. 10.1038/d41586-019-00766-x 30837734

[27] Dewitt B, Fischhoff B, Sahlin N-E. “Moral machine” experiment is no basis for policymaking. Nature. 2019;567(7746):31. doi: 10.1038/d41586-019-00766-x 30837734

[28] Bostyn DH, Sevenhant S, Roets A. Of Mice, Men, and Trolleys: Hypothetical Judgment Versus Real-Life Behavior in Trolley-Style Moral Dilemmas. Psychol Sci. 2018;29(7):1084–93. doi: 10.1177/0956797617752640 29741993

[29] Fried BH. What Does Matter? The Case for Killing the Trolley Problem (Or Letting It Die). Philosl Q. 2012;62(248):505–29. doi: 10.1111/j.1467-9213.2012.00061.x

[30] Goodall NJ. Away from Trolley Problems and Toward Risk Management. Applied Artificial Intelligence. 2016;30(8):810–21. doi: 10.1080/08839514.2016.1229922

[31] Hübner D, White L. Crash Algorithms for Autonomous Cars: How the Trolley Problem Can Move Us Beyond Harm Minimisation. Ethic Theory Moral Prac. 2018;21(3):685–98. doi: 10.1007/s10677-018-9910-x

[32] Müller VC. Autonomous Killer Robots are Probably Good News. In: Nucci ED, Sio FS de, editors. Drones and Responsibility: Legal, Philosophical and Socio-Technical Perspectives on Remotely Controlled Weapons. Routledge; 2016. p. 67–81.

[33] Grossman D. On killing: the psychological cost of learning to kill in war and society. Revised ed. ed. New York: Back Bay Books; 2009.

[34] Bigman YE, Gray K. People are averse to machines making moral decisions. Cognition. 2018;181:21–34. doi: 10.1016/j.cognition.2018.08.003 30107256

[35] EU. Resolution on autonomous weapon systems (2018/2752). European Parliament; 2018. Available: https://oeil.secure.europarl.europa.eu/oeil/popups/ficheprocedure.do?lang=en&reference=2018/2752(RSP)

[36] Coeckelbergh M. Drones, morality, and vulnerability: two arguments against automated killing. In: Custers B, editor. The Future of Drone Use: Opportunities and Threats from Ethical and Legal Perspectives. The Hague: T.M.C. Asser Press; 2016. p. 229–37. doi: 10.1007/978-94-6265-132-6_12

[37] Santoni de Sio F. Killing by Autonomous Vehicles and the Legal Doctrine of Necessity. Ethic Theory Moral Prac. 2017;20(2):411–29. doi: 10.1007/s10677-017-9780-7

[38] Scheutz M, Malle BF. May machines take lives to save lives? Human perceptions of autonomous robots (with the capacity to kill). In: Galliott J, MacIntosh D, Ohlin JD, editors. Lethal autonomous weapons: re-examining the law and ethics of robotic warfare. Oxford University Press; 2020.

[39] Ganesh MI. The ironies of autonomy. Humanit Soc Sci Commun. 2020;7(1). doi: 10.1057/s41599-020-00646-0

[40] Marres N. What If Nothing Happens? Street Trials of Intelligent Cars as Experiments in Participation. In: Maasen S, Dickel S, Schneider C, editors. TechnoScienceSociety: Technological Reconfigurations of Science and Society. Cham: Springer International Publishing; 2020. p. 111–30. doi: 10.1007/978-3-030-43965-1_7

[41] Stilgoe J, Mladenović M. The politics of autonomous vehicles. Humanit Soc Sci Commun. 2022;9(1). doi: 10.1057/s41599-022-01463-3 PMC9395839 36032807

[42] Martinho A, Herber N, Kroesen M, Chorus C. Ethical issues in focus by the autonomous vehicles industry. Transport Reviews. 2021;41(5):556–77. doi: 10.1080/01441647.2020.1862355

[43] Iapaolo F. The system of autono‑mobility: computer vision and urban complexity-reflections on artificial intelligence at urban scale. AI Soc. 2023;38(3):1111–22. doi: 10.1007/s00146-022-01590-0 37215367 PMC10165562

[44] Arfini S, Spinelli D, Chiffi D. Ethics of Self-driving Cars: A Naturalistic Approach. Minds & Machines. 2022;32(4):717–34. doi: 10.1007/s11023-022-09604-y

[45] Geisslinger M, Poszler F, Betz J, Lütge C, Lienkamp M. Autonomous Driving Ethics: from Trolley Problem to Ethics of Risk. Philos Technol. 2021;34(4):1033–55. doi: 10.1007/s13347-021-00449-4

[46] Stilgoe J. How can we know a self-driving car is safe? Ethics Inf Technol. 2021;23(4):635–47. doi: 10.1007/s10676-021-09602-1

[47] Blackstone A. Principles of sociological inquiry: qualitative and quantitative methods. Washington, D.C.: Saylor Foundation; 2012.

[48] Marshall MN. Sampling for qualitative research. Fam Pract. 1996;13(6):522–5. doi: 10.1093/fampra/13.6.522 9023528

[49] Saunders B, Sim J, Kingstone T, Baker S, Waterfield J, Bartlam B, et al. Saturation in qualitative research: exploring its conceptualization and operationalization. Qual Quant. 2018;52(4):1893–907. doi: 10.1007/s11135-017-0574-8 29937585 PMC5993836

[50] Campbell R, Kumar V. Moral Reasoning on the Ground. Ethics. 2012;122(2):273–312. doi: 10.1086/663980

[51] Kahane G. The armchair and the trolley: an argument for experimental ethics. Philos Stud. 2013;162(2):421–45. doi: 10.1007/s11098-011-9775-5 23316090 PMC3540347

[52] Knobe J, Buckwalter W, Nichols S, Robbins P, Sarkissian H, Sommers T. Experimental philosophy. Annu Rev Psychol. 2012;63:81–99. doi: 10.1146/annurev-psych-120710-100350 21801019

[53] Braun V, Clarke V. Using thematic analysis in psychology. Qualitative Research in Psychology. 2006;3(2):77–101. doi: 10.1191/1478088706qp063oa

[54] Guest G, MacQueen K, Namey E. Applied Thematic Analysis. SAGE Publications, Inc.; 2012. doi: 10.4135/9781483384436

[55] Mayring P. Qualitative content analysis: theoretical foundation, basic procedures and software solution. Klagenfurt: SSOAR; 2014. Available: https://www.ssoar.info/ssoar/handle/document/39517

[56] Milford SR, Malgir BZ, Elger BS, Shaw DM. “All things equal”: ethical principles governing why autonomous vehicle experts change or retain their opinions in trolley problems-a qualitative study. Front Robot AI. 2025;12:1544272. doi: 10.3389/frobt.2025.1544272 40496372 PMC12148897

[57] Milford SR, Simone Elger B, Shaw D. Bearing the weight: A qualitative study on expert views on integrating ethics in autonomous vehicles. Transportation Research Interdisciplinary Perspectives. 2024;25:101096. doi: 10.1016/j.trip.2024.101096

[58] Searle JR. Minds, brains, and programs. Behav Brain Sci. 1980;3(3):417–24. doi: 10.1017/s0140525x00005756

[59] Himma KE. Artificial agency, consciousness, and the criteria for moral agency: what properties must an artificial agent have to be a moral agent? Ethics Inf Technol. 2008;11(1):19–29. doi: 10.1007/s10676-008-9167-5

[60] Sparrow R. Why machines cannot be moral. AI & Soc. 2021;36(3):685–93. doi: 10.1007/s00146-020-01132-6

[61] Bonnefon JF. The car that knew too much: Can a machine be moral? Cambridge: MIT Press; 2021.

[62] European Commission. Statement on artificial intelligence, robotics and “autonomous” systems. Publications Office of the European Union; 2018. Available: https://data.europa.eu/doi/10.2777/531856

[63] Frank D-A, Chrysochou P, Mitkidis P, Ariely D. Human decision-making biases in the moral dilemmas of autonomous vehicles. Sci Rep. 2019;9(1):13080. doi: 10.1038/s41598-019-49411-7 31511560 PMC6739396

[64] Cunneen M, Mullins M, Murphy F, Shannon D, Furxhi I, Ryan C. Autonomous Vehicles and Avoiding the Trolley (Dilemma): Vehicle Perception, Classification, and the Challenges of Framing Decision Ethics. Cybernetics and Systems. 2019;51(1):59–80. doi: 10.1080/01969722.2019.1660541

[65] Hang P, Lv C, Xing Y, Huang C, Hu Z. Human-Like Decision Making for Autonomous Driving: A Noncooperative Game Theoretic Approach. IEEE Trans Intell Transport Syst. 2021;22(4):2076–87. doi: 10.1109/tits.2020.3036984

[66] Contissa G, Lagioia F, Sartor G. The Ethical Knob: ethically-customisable automated vehicles and the law. Artif Intell Law. 2017;25(3):365–78. doi: 10.1007/s10506-017-9211-z

[67] Formosa P. Autonomous vehicles and ethical settings: who should decide? In: Jenkins R, Cerny D, Hribek T, editors. Autonomous Vehicle Ethic. Oxford University Press; 2022.

[68] Awad E, Bonnefon J-F, Shariff A, Rahwan I. The Thorny Challenge of Making Moral Machines: Ethical Dilemmas with Self-Driving Cars. NIM Marketing Intelligence Review. 2019;11(2):42–7. doi: 10.2478/nimmir-2019-0015

[69] Shaw DM, Schneble CO. Advance Car-Crash Planning: Shared Decision Making between Humans and Autonomous Vehicles. Sci Eng Ethics. 2021;27(6):75. doi: 10.1007/s11948-021-00358-x 34907470

[70] von Eschenbach WJ. Transparency and the Black Box Problem: Why We Do Not Trust AI. Philos Technol. 2021;34(4):1607–22. doi: 10.1007/s13347-021-00477-0

[71] Galliott J. Humans, Autonomous Systems, and Killing in War. Research Anthology on Military and Defense Applications, Utilization, Education, and Ethics. IGI Global; 2021. p. 240–57. doi: 10.4018/978-1-7998-9029-4.ch013

[72] Winfield AFT, Jirotka M. The case for an ethical black box. In: Gao Y, Fallah S, Jin Y, Lekakou C, editors. Towards Autonomous Robotic Systems. Cham: Springer International Publishing; 2017. p. 262–73. doi: 10.1007/978-3-319-64107-2_21

[73] Umbrello S, Yampolskiy RV. Designing AI for Explainability and Verifiability: A Value Sensitive Design Approach to Avoid Artificial Stupidity in Autonomous Vehicles. Int J of Soc Robotics. 2021;14(2):313–22. doi: 10.1007/s12369-021-00790-w

[74] Coeckelbergh M. Artificial Intelligence, Responsibility Attribution, and a Relational Justification of Explainability. Sci Eng Ethics. 2020;26(4):2051–68. doi: 10.1007/s11948-019-00146-8 31650511 PMC7417397

[75] Santoni de Sio F, van den Hoven J. Meaningful Human Control over Autonomous Systems: A Philosophical Account. Front Robot AI. 2018;5:15. doi: 10.3389/frobt.2018.00015 33500902 PMC7806098

[76] Denzin NK, Lincoln YS, Giardina MD, Cannella GS. The SAGE handbook of qualitative research. 6th ed. Los Angeles, London, New Delhi, Singapore, Washington DC, Melbourne: SAGE Publications, Inc; 2023.

[77] Miles MB, Huberman AM. Qualitative data analysis: an expanded sourcebook. 2nd ed. Thousand Oaks, Calif.: SAGE Publications, Inc; 1994.

